# Revealing the dynamic changes of metabolites and molecular mechanisms of chlorogenic acid accumulation during the leaf development of *Vaccinium dunalianum* based on multi-omic analyses

**DOI:** 10.3389/fpls.2024.1440589

**Published:** 2024-10-31

**Authors:** Anmian Zhang, Jiaxin Liu, Weicheng Li, Lihong Yang, Wenjin Duan, Ping Zhao, Zhiyu Pu, Yong Ding

**Affiliations:** ^1^ Key Laboratory for Forest Resources Conservation and Utilization in the Southwest Mountains of China, Ministry of Education, Southwest Forestry University, Kunming, China; ^2^ College of Biological Science and Food Engineering, Southwest Forestry University, Kunming, China; ^3^ Department of Economic Plants and Biotechnology, Yunnan Key Laboratory for Wild Plant Resources, Kunming Institute of Botany, Chinese Academy of Sciences, Kunming, China

**Keywords:** *Vaccinium dunalianum*, Quezui tea, chlorogenic acid (CGA), transcriptomics, proteomics, non-targeted metabolomics, network pharmacology

## Abstract

*Vaccinium dunalianum*, a medicinal plant, is utilized for Quezui Tea production from its leaf buds and young leaves. Despite prior research on *V. dunalianum* revealing several medicinal compounds, the comprehensive variations in metabolites during its growth and development, along with the molecular mechanisms underlying high chlorogenic acid (CGA) yield, remain unclear. Through a joint analysis of transcriptomics and proteomics, our study first identified 15 key structural genes and 3 transcription factors influencing CGA biosynthesis in *V. dunalianum*, offering new evidence to understand its regulatory network. Furthermore, non-targeted metabolomics analysis provides the first extensive report on the metabolic profile of *V. dunalianum*, furnishing a valuable dataset for deeper exploration of its nutritional and medicinal value, and the development of a quality evaluation system for its product Quezui Tea. This study offers the most comprehensive molecular information on *V. dunalianum*, marking a significant step toward understanding and enhancing the plant’s potential for medicinal and nutritional applications. Additionally, this study also reveals *V. dunalianum* holds promise as a natural antioxidant source for functional foods, providing data support for network pharmacology.

## 1 Introduction

The *Vaccinium dunalianum* Wight, an evergreen shrub belonging to the *Vaccinium* genus in the Ericaceae family, is native to the Yunnan region of China, where its leaf buds and young leaves have been processed into a tea beverage known as Quezui Tea since the Ming Dynasty. *V. dunalianum* is rich in various polyphenolic antioxidants and serves as a significant natural dietary source of phenolic compounds (Gao et al., 2022). It mainly comprises arbutin, 6’-*O*-caffeoylarbutin, and chlorogenic acid (CGA), along with a range of flavonoids and phenolic acid compounds (Cheng et al., 2022). Recent reports indicate that Quezui Tea possesses a broad spectrum of medicinal properties, with therapeutic effects including the promotion of liver detoxification, weight loss, blood circulation improvement, and reduction of blood glucose and lipid levels (Zhang et al., 2022; Yang et al., 2023). Additionally, the entire *V. dunalianum* plant is harnessed for the treatment of various conditions, including alleviating rheumatoid arthritis pain, soothing sore throats, and addressing constipation. *V. dunalianum* exhibits significant economic value in medicinal, dietary, and chemical industries, leading to increased recognition of *V. dunalianum* as a distinctive resource plant, thus prompting in-depth research on this plant. Currently, research on *V. dunalianum* primarily focuses on several key areas, including the extraction and functional analysis of medicinally active ingredients (Wang et al., 2021b; Zhao et al., 2021; Gao et al., 2022; Yang et al., 2023), the isolation, purification, and quantification of CGA and other bioactive substances (Luo et al., 2015; Li et al., 2018), and the safety assessment of its product Quezui Tea (Wang et al., 2021a). Notably, CGA as a crucial secondary metabolite, is highly enriched in the leaf buds of *V. dunalianum*, accounting for 76 mg/g of dry weight (Luo et al., 2015). The high yield characteristics of CGA and various tea polyphenols in the leaf buds and young leaves of *V. dunalianum* contribute significantly to the nutritional and medicinal value of Quezui Tea. Currently, no research has been reported on the molecular mechanisms for the high production traits of CGA in *V. dunalianum* or the composition and variations of metabolites during its leaf development. The composition and content of metabolites in the leaf buds and young leaves of *V. dunalianum* have a profound impact on the flavor, taste, and nutritional value of Quezui Tea, making them crucial indicators for evaluating the quality of Quezui Tea. These metabolite compositions and abundances are influenced by various factors, with growth and development being one of the most important factors.

With the rapid advancement of high-resolution mass spectrometry and high-throughput sequencing technologies, along with the continuous refinement of machine learning and data mining methods, multi-omics joint analysis is widely applied across various fields in life sciences (Reel et al., 2021; Das et al., 2023; Li et al., 2023; Wang et al., 2023). Actually, resolving questions in life sciences often requires multidimensional information for characterization and validation. Multi-omics integrated analysis can provide additional evidence from multiple dimensions, shedding light on potential regulatory networks and mechanisms in biological systems (Jung et al., 2020; Zhang et al., 2023). Ge et al. conducted multi-omics analyses to propose the formation patterns of wax and cutin polyester network for pepper cuticle (Ge et al., 2022). Lou et al. elucidated the amino acid biosynthetic pathway and identified the key genes in *Torreya grandis* (Lou et al., 2022). Sun et al. revealed the changes of differentially accumulated metabolites and abundant proteins of *Scutellaria baicalensis* in different growth years and provided a scientific reference for determining the optimal picking period and clinically rational use of *S. baicalensis* (Sun et al., 2023). In addition, similar studies have been applied to many species such as kiwifruit (Shu et al., 2023), sweet orange (Xiong et al., 2023), blueberry (Li et al., 2023), yam (Cao et al., 2023), rice (Yang et al., 2022), tomato (Li et al., 2020), etc. However, the dynamic changes in the metabolic profile and the molecular mechanisms underlying differential accumulation of CGA during *V. dunalianum* leaf development have not been reported.

Herein, our study utilized multi-omics analysis to uncover molecular mechanisms behind high CGA yield in *V. dunalianum* and examined dynamic metabolic profile changes during *V. dunalianum* leaf development. The findings support Quezui Tea’s nutritional value and quality identification, aiding the industrialization of *V. dunalianum* plant resource application through molecular breeding.

## 2 Materials and methods

### 2.1 Plant materials

Leaf buds (VdLB), young leaves (VdYL), and mature leaves (VdML) of *V. dunalianum* were collected from Wuding County, Yunnan Province, China (25°45’N; 102°17’E) in April 2022. Flower buds (VdFB) were collected from the same location as above. The plant samples were rapidly frozen in liquid nitrogen on-site and later stored at −80°C in the laboratory for subsequent experiments. The leaves of each developmental stage comprised six independent biological replicates for non-targeted metabolomics experiments, and three independent biological replicates for Illumina RNA-Sequencing (RNA-Seq), quantitative real time PCR (qRT-PCR) analysis, quantitative proteomics, and CGA content determination by High-Performance Liquid Chromatography (HPLC). The tissue samples from VdLB, VdML, and VdFB were used for third-generation full-length transcriptome sequencing.

### 2.2 Analysis of CGA content across three developmental stages of *V. dunalianum* leaves

The content of chlorogenic acid was determined by HPLC technology (Luo et al., 2015), with certain refinements implemented. The samples from VdLB, VdYL, and VdML were steamed for 5 minutes, followed by natural drying in the shade until a constant weight was achieved. Subsequently, they were pulverized into powder and sieved through a 40-mesh sieve. A 0.1 g portion of the resulting powder was weighed and dissolved in 1.6 mL of 73% methanol. Ultrasonic extraction was performed in a water bath for 19 minutes, followed by centrifugation at 5000 rpm for 2 minutes to obtain the supernatant for further processing. This extraction process was repeated three times with 73% methanol. From each obtained supernatant, 1 mL extract was collected, thoroughly mixed, and subsequently subjected to filtration through a 0.45 μm microporous membrane for CGA analysis.

Analytical standard CGA (CAS Number: 327-97-9) was obtained from Shanghai Yuanye Bio-Technology Co., Ltd. A 3 mg CGA standard was accurately weighed and dissolved in 2 mL of 73% methanol to create a 1.5 mg/mL stock solution. This stock solution was then subjected to a sequential gradient dilution process, generating seven distinct concentrations: 1.5 mg/mL, 0.75 mg/mL, 0.375 mg/mL, 0.1875 mg/mL, 0.09375 mg/mL, 0.046875 mg/mL, and 0.0234375 mg/mL. The resulting CGA standard solutions were stored at 4°C for stability and designated for subsequent HPLC analyses.

HPLC analyses of CGA were conducted using an Agilent 1260 Series HPLC system, and data processing was performed using OpenLab CDS software. The quantification of CGA content was executed employing a VWD detector, with the detection wavelength configured at 280 nm. The separation was made on the CAPCELL PAK C18 MG (4.60×250mm, 5μm) column. The mobile phase consisted of two components: acetic acid 0.1% in water (solution A) and 100% methanol (solution B). The composition of the gradient was (A: B), 95:5 at 0 min, 95: 5 at 5 min, 85:15 at 6 min, 35:65 at 25 min, 5:95 at 30 min, 95:5 at 35 min. The temperature of the chromatographic column was set at 25°C with a variation of ±5°C. For each sample and standard solution, an injection volume of 10 μL was employed.

### 2.3 Transcriptomics analysis

#### 2.3.1 Transcriptome library preparation and sequencing

The total RNA of VdLB, VdYL, and VdML was separately extracted using the Trizol Reagent (Invitrogen Life Technologies), after which the Illumina cDNA libraries from single tissue were sequenced using a NovaSeq 6000 platform (Illumina) by Shanghai Personal Biotechnology Co., Ltd. The total RNA from three tissues: VdLB, VdML, and VdFB was separately extracted and subsequently equally mixed to construct PacBio cDNA libraries that were sequenced with a Pacific Biosciences Sequel sequencing instrument.

#### 2.3.2 Analysis of PacBio data and Illumina data

SMRT sequencing data were processed using the SMRTlink 6.0 software. Circular consensus sequences (CCS) were generated and classified into full-length non-chimera (FLNC) and non-full-length (nFL) reads. The isoform-level clustering (ICE) algorithm was applied to cluster identical transcripts, forming consensus sequences. These served as the reference transcriptome, corrected using nFL reads to produce polished consensus sequences. The filtered full-length transcripts underwent comprehensive functional annotation through various databases, including Nr, Swiss-Prot, GO, eggNOG, PFAM, and KEGG.

The clean reads from Illumina sequencing were mapped to the reference full-length transcriptome using Bowtie2. RSEM (v2.15) statistics were employed to compare the read count values for each gene as the original gene expression, and the expression was standardized using fragments per kilobase per million (FPKM). Differential expression genes(DEGs) analysis was performed using DESeq (Wang et al., 2010), and the p-values < 0.05 and |log_2_FoldChange| >1 were used as thresholds for significant differential expression. DEGs underwent KEGG pathway enrichment (clusterprofiler), expression trend analysis (Mfuzz), and GO enrichment (topGO, p < 0.05). Visualization used REVIGO for GO analysis and chiplot for circular heatmaps (https://www.chiplot.online/).

### 2.4 Proteomic analysis during leaf development

The 4D label-free quantitative proteomic detection of VdLB, VdYL, and VdML was entrusted to Jingjie Biotechnology Co., Ltd. The experimental procedure was carried out in a way that was described in a previous study with minor modifications (Ding et al., 2023). The leaf tissue samples were ground, lysed, and centrifuged to collect the supernatant. The protein concentration of the supernatant was measured and then digested with trypsin to produce peptides. The Peptide segments were subjected to chromatographic analysis employing a nanoElute ultrahigh-performance liquid chromatography (LC) system. After the chromatographic separation, the eluted peptides were introduced into a capillary ion source for ionization. The resulting ionized peptides, including both the parent ions and their associated secondary fragments, were subjected to analysis using high-resolution time-of-flight mass spectrometry (TOF-MS). Data acquisition was performed in parallel accumulation-serial fragmentation (PASEF) mode. The MS/MS data obtained were subsequently subjected to analysis using the MaxQuant search engine (version 1.6.15.0).

The fold change was determined by calculating the ratio of the mean values of quantitative biological replicates for each protein in the compared sample pairs. Differential expression proteins (DEPs) were selected based on the criteria of p-value < 0.05 and |log_2_FoldChange| >1. Volcano plots for DEPs between comparison groups were generated using the ggplot2 package in R, and nine quadrant plots depicting the relationship between DEPs and DEGs were created.

### 2.5 Non-targeted metabolomics analysis during leaf development

The leaf samples, sent to Personalbio in Shanghai for the determination of metabolites, followed a method outlined in a prior study with slight modifications (Wang et al., 2021). The 80 mg sample was flash-frozen in liquid nitrogen, ground into powder, and metabolites were extracted with a 1000 μL mixture of methanol/acetonitrile/water, followed by vortexing and two rounds of low-temperature sonication, each lasting 30 minutes. Samples were incubated at −20°C for 60 minutes, followed by centrifugation at 14,000 g for 20 minutes at 4°C. The supernatant was then vacuum dried, reconstituted in a 1:1 mixture of acetonitrile and water, and subjected to a second centrifugation. LC-MS analysis was performed using an Agilent 1290 Infinity LC UHPLC system. The chromatographic separation was conducted on a C18 column (2.1 × 100 mm, 1.7 μm) with a flow rate of 0.5 mL/min, an injection volume of 2 μL, and a column temperature of 25°C. To further ensure data reliability, we prepared four quality control (QC) samples by pooling 10 μL from each sample and subsequently analyzed them alongside the other samples. The AB Triple TOF 6600 mass spectrometer (AB SCIEX, Massachusetts, USA) was utilized to obtain primary and secondary spectra of the samples in positive ion and negative ion modes using electrospray ionization (ESI).

The in-depth analysis of non-targeted metabolomics is conducted using methods such as Zhang et al (Zhang et al., 2022). Briefly, differentially accumulated metabolites (DAMs) were assessed using the variable importance in projection (VIP) scores obtained from the orthogonal partial least-squares discriminant analysis (OPLS-DA) model, applying thresholds of VIP ≥ 1, |log_2_FoldChange| >1, and p-value ≤ 0.05. A clustering heatmap for the DAMs was created using the R pheatmap package.

### 2.6 Quantitative real time PCR detection

Total RNA of VdLB and VdML were reverse-transcribed to synthesize cDNAs using Vayzme HiScript II 1st Strand cDNA Synthesis kit with gDNA wiper. Primer of selected unigenes was designed using Primer-BLAST (https://www.ncbi.nlm.nih.gov/tools/primer-blast/) (**Supplementary Table S1**). qRT-PCR was performed using PerfectStart Green qPCR SuperMix kit (TransGen). The Perfect Start Green qPCR Super Mix kit (transgenic) was used for qRT-PCR, and the reaction system was 20 μL. The reaction system included 1 μL cDNA template, 10 μL 2 × ChamQ Blue Universal SYBR qPCR Master Mix, 0.4 μL of each 10 μM forward and reverse primers, and 8.2 μL ddH_2_O. The qRT-PCR amplification conditions were as follows: perform pre-denaturation at 95°C for 30 s, denaturation at 95°C for 5 s, and annealing at 60°C for 15 s. After amplification, a melting curve analysis from 65-95°C was performed to determine the product specificity. All qRT-PCR experiments were performed with three technical and 3 biological repeats on a CFX96 Real-Time PCR Detection System. The stable internal reference gene (Initiation factor-4A, EIF-4A-2), previously reported in *V. dunalianum* studies (Shao et al., 2022), was utilized as the reference gene, and the relative expression level was calculated employing the 2^-ΔΔCt^ method.

### 2.7 Statistical analysis

Student’s t-test in R software was used to analyze the differences in CGA content between any two samples of *V. dunalianum* leaves, and the results were considered statistically significant at p < 0.05. Principal component analysis (PCA) and correlation analysis were conducted in R.

## 3 Results and discussion

### 3.1 Determination of CGA content at three development stages of *V. dunalianum* leaves

**Figure 1:**
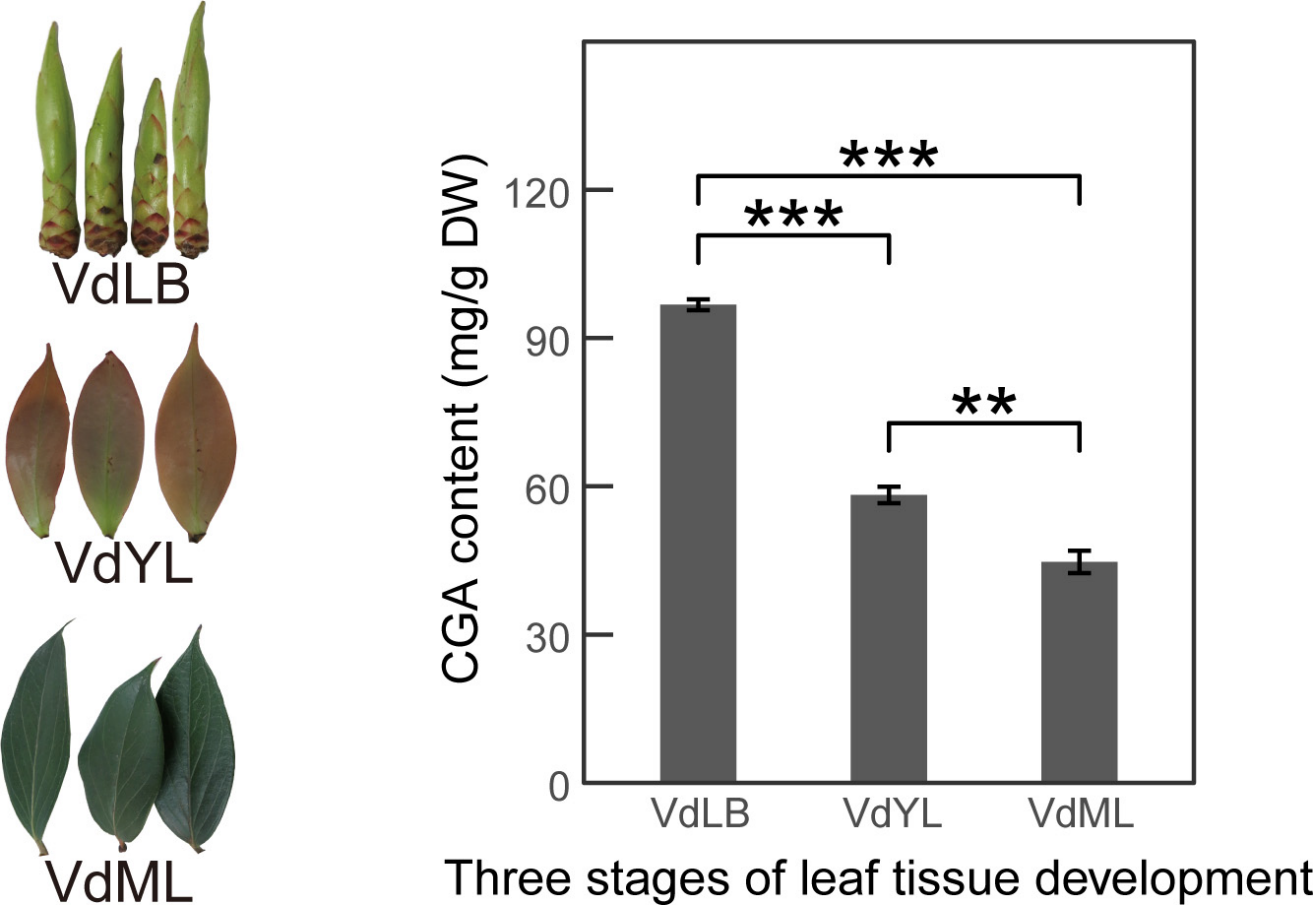
Analysis of CGA content in *V. dunalianum* leaves at different development stages. CGA content in the three developmental stages of *V. dunalianum* leaves (VdLB, VdYL, VdML). The data represent the mean values ± standard deviation of three biological replicates. Asterisks denote statistical significance based on Student’s t-test (**: *P* < 0.01; ***: *P* < 0.001).

The content of CGA in *V. dunalianum* leaves was found to decrease gradually with their growth and development, as determined by quantitative analysis using HPLC technology. Specifically, CGA content in VdLB, VdYL, and VdML was quantified at 97.64 ± 1.10 mg/g, 58.26 ± 1.68 mg/g, and 44.71 ± 2.26 mg/g, respectively (**Figure 1**). Significant differences in CGA content were observed between any two developmental stages (P<0.01), with VdLB exhibiting a notably higher CGA content compared to VdYL and VdML (P<0.001).

The CGA, as a potent antioxidant, with its ability to counteract the generation of free radicals and oxidative damage in plants, as well as its antimicrobial and antiviral properties, contributes to enhancing *V. dunalianum* adaptability and survival. These effects play a crucial role in regulating the formation and expansion of leaves during *V. dunalianum* leaf development. During the developmental process of *V. dunalianum* leaves, the susceptibility of young tissues to oxidative damage and environmental stress is notably higher compared to mature tissues, which accounts for the high production of CGA in VdLB.

### 3.2 Multi-omics quality control

**Figure 2:**
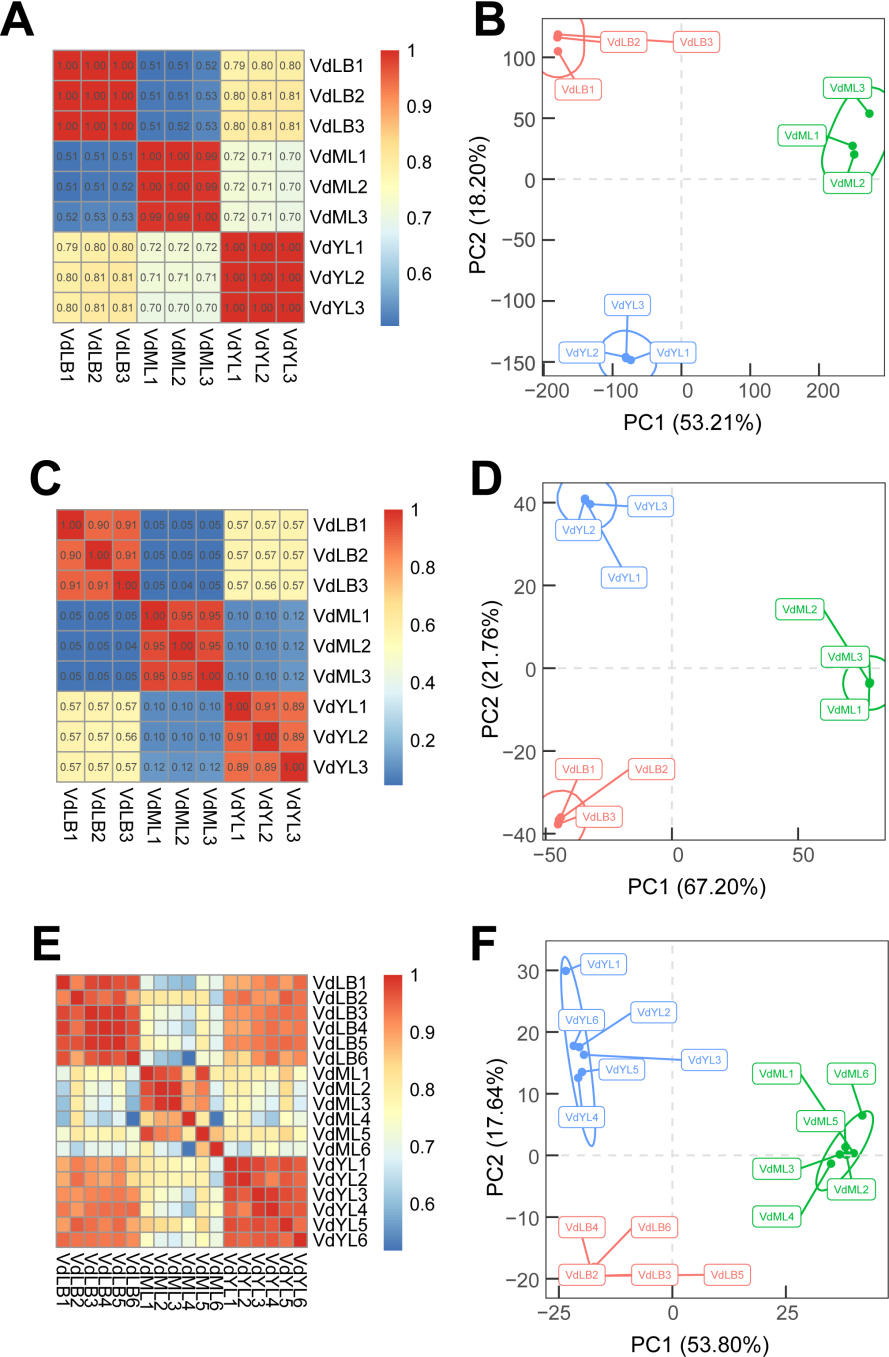
Analysis of correlation and PCA of transcriptomics, proteomics, and non-targeted metabolomics in *V. dunalianum* leaves at three development stages. **(A)** Correlation analysis of transcriptomics. **(B)** PCA analysis of transcriptomics. **(C)** Correlation analysis of proteomics. **(D)** PCA analysis of proteomics. **(E)** Correlation analysis of metabolomics. **(F)** PCA analysis of metabolomics.

The samples from VdLB, VdYL, and VdML were utilized for RNA-Seq, quantitative proteomic, and untargeted metabolomic. The total ion chromatograms (TIC) of QC samples of untargeted metabolomics in both positive and negative ion modes indicate that the system remained stable throughout the experimental process, enabling in-depth analysis of metabolites (**Supplementary Figure S1**). The unsupervised PCA and correlation analysis conducted in this study demonstrate the reliability of the data obtained from the three omics datasets (**Figure 2**). The results of the correlation and PCA analyses of transcriptome expression profiles showed that samples from different development stages were distinctly separated, while the biological replicate samples clustered closely together, indicating a high correlation (**Figures 2A, B**). This suggested that leaf tissues at different developmental stages exhibited distinct patterns of gene expression. Furthermore, the high consistency among the biological replicate samples further verified the reliability of the subsequent analysis results. The PCA and correlation analysis of protein expression profiles at different developmental stages of *V. dunalianum* leaves showed that the biological replicate samples were clustered together at the same developmental stage, and their protein expression patterns were highly correlated (**Figures 2C, D**). Concurrently, there were differences in protein abundance among samples from different developmental stages, reflecting that the proteins and their regulatory networks underwent a dynamic reconstruction process during the growth and development of *V. dunalianum* leaves. The results of PCA and correlation analysis were similar to the results of the corresponding analysis of transcriptome and proteome. The difference of metabolite abundance between biological repeat samples was small, and the samples at different development stages were obviously separated, and the difference was large (**Figures 2E, F**).

Thus, we conclude that there is a close relationship between the dynamic accumulation of metabolites and the regulation of gene expression in *V. dunalianum* leaves. Indeed, in plants, the production and accumulation of metabolites are largely influenced by external environmental factors and inherent developmental processes. Specifically, the developmental stage of plant tissues significantly influences the accumulation of metabolites.

### 3.3 PacBio data analysis

A total of 19.39 gigabases (Gb) of polymerase reads were generated, with the overall number of polymerase reads reaching 567,000, through PacBio SMRT sequencing. The average polymerase read length and polymerase read N50 were 34,201 base pairs(bp) and 57,704 bp, respectively. After removing the adapters, a total of 10 747,907 subreads (18.59 Gb) were generated, with an average length of 1,730 bp and an N50 of 2,533 bp. Then, a total of 470,827 CCS reads were extracted, and max_length, mean_length, and N50 were 14,910 bp, 2,454 bp and 2,952 bp, respectively. Subsequently, 333,916 FLNC sequences were obtained with an average length of 2,362 bp and an N50 of 2,700 bp. After filtering low-quality and ambiguous reads, 177,860 polished high-quality consensus reads (transcripts) were generated with a max_length of 13,651 bp, mean_length of 2,399 bp, and an N50 of 2,844 bp. After redundant sequences were removed, a refined dataset of 93,458 unigenes, totaling 247,173,734 nucleotides, was obtained. This reduction improves data clarity and reliability, providing a solid foundation for further analysis.

The annotation, pathway, and functional categorization of the unigenes were thoroughly analyzed using the KEGG, GO, PFAM, Swiss-Prot, eggNOG, and Nr databases. The results showed that 85,200 unigenes were annotated in at least one database, accounting for 91.16% of the total number of unigenes (**Table 1**). Additionally, 8,258 unigenes, representing 8.84% of all unigenes, lacked functional annotation information, potentially encompassing a substantial number of *V. dunalianum*-specific gene sequences and novel transcripts.

**Table 1:** Summary of unigenes annotation results.

Databases	Total unigenes	Annotated unigenes	Percent(%)
NR	93,458	84,372	90.28
GO	93,458	55,841	59.75
KEGG	93,458	40,325	43.15
Pfam	93,458	45,343	48.52
eggNOG	93,458	79,329	84.88
Swiss-Prot	93,458	69,337	74.19
In all databases	93,458	19,847	21.24
At least one database	93,458	85,200	91.16

So far, genomic information for *V. dunalianum* has not been unveiled, resulting in a lack of reliable genetic data, which severely hampers the precise comprehension of genetic diversity and overall expression patterns in *V. dunalianum*. In the absence of genomic information, full-length transcriptome sequencing is the valid option to study the transcriptomes of most organisms to date (Deng et al., 2022). Although Illumina RNA-Seq, *de novo* assembly of the transcriptome based on second-generation sequencing (NGS), has provided valuable insights into the dynamic accumulation of metabolites and the regulation of gene expression, there are still some disadvantages, such as a short sequencing length, the incomplete or inaccurate splicing (Ari and Arikan, 2016; Yan et al., 2022). Moreover, the genomic information of *V. dunalianum* has not been sequenced yet. Herein, the full-length transcriptome database obtained in this study was used as a reference transcriptome for RNA-Seq and proteomic analysis of leaf samples at three developmental stages of *V. dunalianum*, which greatly improved the quality of the omics data and the reliability of the analysis results. Furthermore, the reference transcriptome will provide a full-length transcriptome data reference for other studies of *V. dunalianum*, which is crucial for future genome annotation and the investigation of gene function in *V. dunalianum*.

### 3.4 Illumina data analysis

#### 3.4.1 Analysis of the RNA-Seq data

Illumina RNA-Seq was performed on *V. dunalianum* leaves at three developmental stages (VdLB, VdYL, and VdML), generating a total of 378,717,722 raw reads. For all samples, the average Q20 and Q30 values for the raw data were 97.45% and 93.02% respectively. Among the total raw reads, 356,096,312 clean reads were derived, and for each sample, the clean reads proportion varied from 93.96% to 94.11%. The clean reads obtained from RNA-Seq were mapped to the reference transcriptome created by PacBio sequencing. Compared to the 93,458 unigenes obtained through PacBio sequencing, the RNA-Seq data across three developmental stages of leaves identified 73,952 unigenes, all of which exhibited expression (FPKM > 0) in at least one of the three leaf developmental stages. The raw RNA-Seq sequence data have been archived in the NCBI Sequence Read Archive (SRA) database with the accession number PRJNA1037676.

The comparison transcriptome analysis of *V. dunalianum* leaves at the three typical developmental stages was conducted to identify DEGs associated with CGA biosynthesis. A total of 8,381, 23,920, and 28,514 DEGs were identified in the “VdYL vs VdLB”, “VdML vs VdYL”, and “VdML vs VdLB” groups, based on the criteria of p-value < 0.05 and |log_2_FoldChange| >1. Remarkably, 3,767 DEGs were found to be present in all three comparison groups (**Supplementary Figure S2**).

#### 3.4.2 KEGG and GO enrichment analysis of DEGs

**Figure 3:**
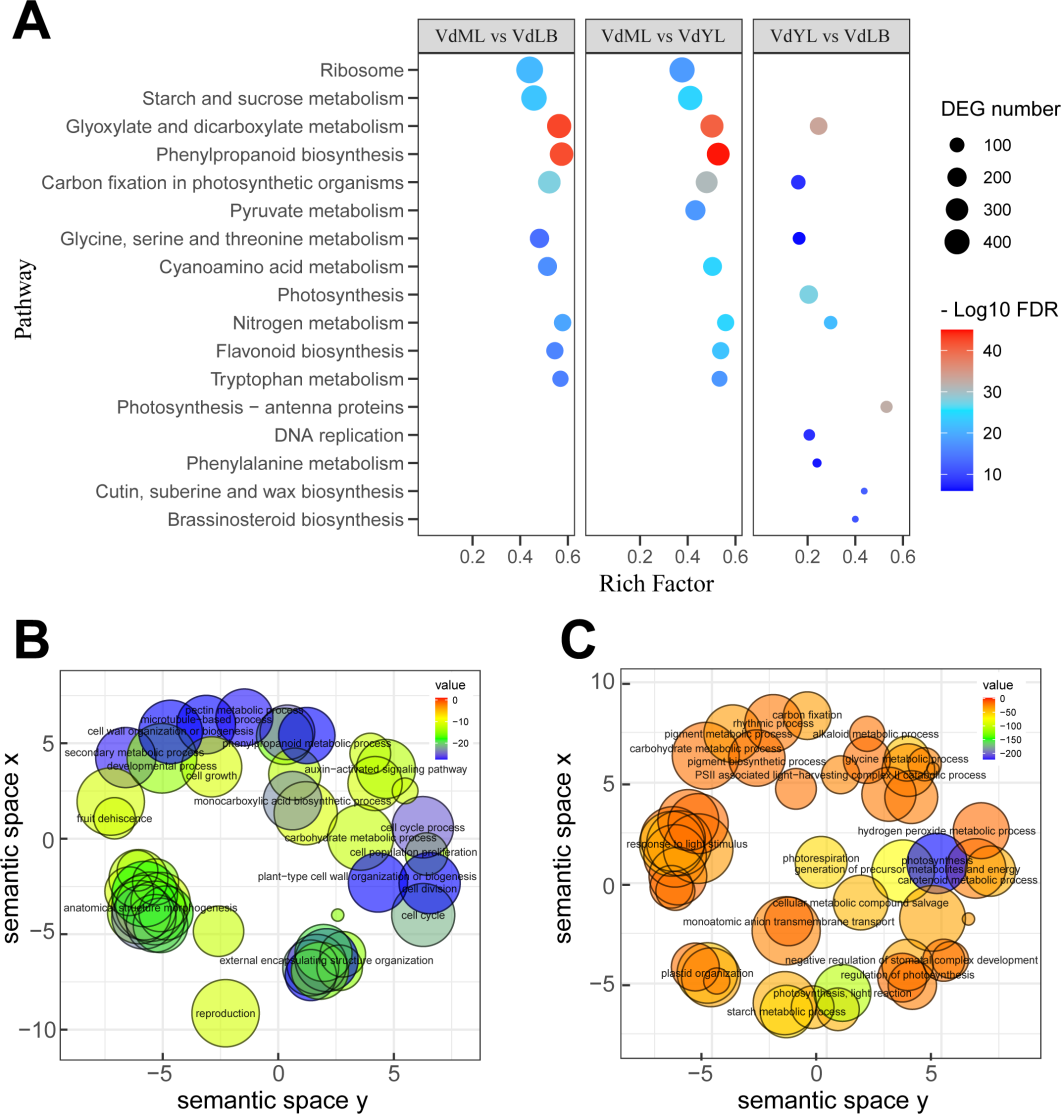
KEGG and GO enrichment analysis of DEGs. **(A)** KEGG enrichment analysis of DEGs in three comparison groups. **(B)** GO analysis of upregulated unigenes in VdML vs VdLB. **(C)** GO analysis of downregulated unigenes in VdML vs VdLB.

To elucidate the specific functional information of DEGs, KEGG enrichment analysis was separately conducted on the DEGs from the three comparison groups to identify the primary pathways in which the DEGs are predominantly involved (**Figure 3A**). The results revealed that, in the “VdML vs VdLB” and “VdML vs VdYL” groups, where DEGs exhibited the most prominent enrichment in the “Glyoxylate and dicarboxylate metabolism” and “Phenylpropanoid biosynthesis” pathways, the two pathways respectively amassed an enrichment of over 300 unigenes in their respective comparison groups. Additionally, in the two comparison groups, several other pathways, such as “Ribosome”, “Starch and sucrose metabolism”, “Flavonoid biosynthesis”, and “Tryptophan metabolism”, among others, also demonstrated substantial enrichment of DEGs. In contrast, the number of DEGs enriched in the “VdYL vs VdLB” group is relatively low.

Due to the highest number of DEGs observed in the “VdML vs VdLB” group, alongside the most substantial variation in CGA content, wherein CGA rapidly accumulates during the VdLB stage, reaching its peak, and subsequently gradually decreases as leaf development progresses, a GO enrichment analysis was conducted on the DEGs from this particular comparison group. This analysis sought a deeper understanding of the molecular mechanisms responsible for the differential accumulation of CGA in *V. dunalianum*. The results indicate that, for upregulated genes, the enriched GO terms primarily include “phenylpropanoid metabolic process”, “secondary metabolic process”, “auxin-activated signaling pathway”, “cell growth”, “cell division”, and “cell population proliferation” (**Figure 3B**). This outcome can be explained from a physiological perspective in plants. During the early stages of leaf development, metabolism is accelerated, and an abundance of auxins is required in leaf buds to facilitate growth and differentiation. Simultaneously, during the growth and development of leaf buds, plants employ secondary metabolic pathways such as the phenylpropanoid metabolism to produce various secondary metabolites, including phenolic acids like CGA and flavonoids, as a response to external environmental stress and pressure. These compounds contribute to antioxidative and antiviral functions, helping the plant withstand biotic and abiotic stressors during leaf development, thus sustaining vigorous growth. As for downregulated genes, the enriched GO terms primarily encompass “photosynthesis”, “regulation of photosynthesis”, “carbon fixation”, “starch metabolic process”, “pigment biosynthetic process”, and “starch metabolic process” (**Figure 3C**). Relative to leaf buds, mature leaves have already established a complete leaf structure, exhibit comparatively vigorous photosynthesis, and feature relatively higher levels of chlorophyll and carotenoids, which are essential for photosynthesis, without the need for the production of substantial amounts of tea polyphenols, as is the case with leaf buds, to ensure the normal progression of their physiological activities.

#### 3.4.3 Analysis of unigenes associated with CGA biosynthesis based on the transcriptome

**Figure 4:**
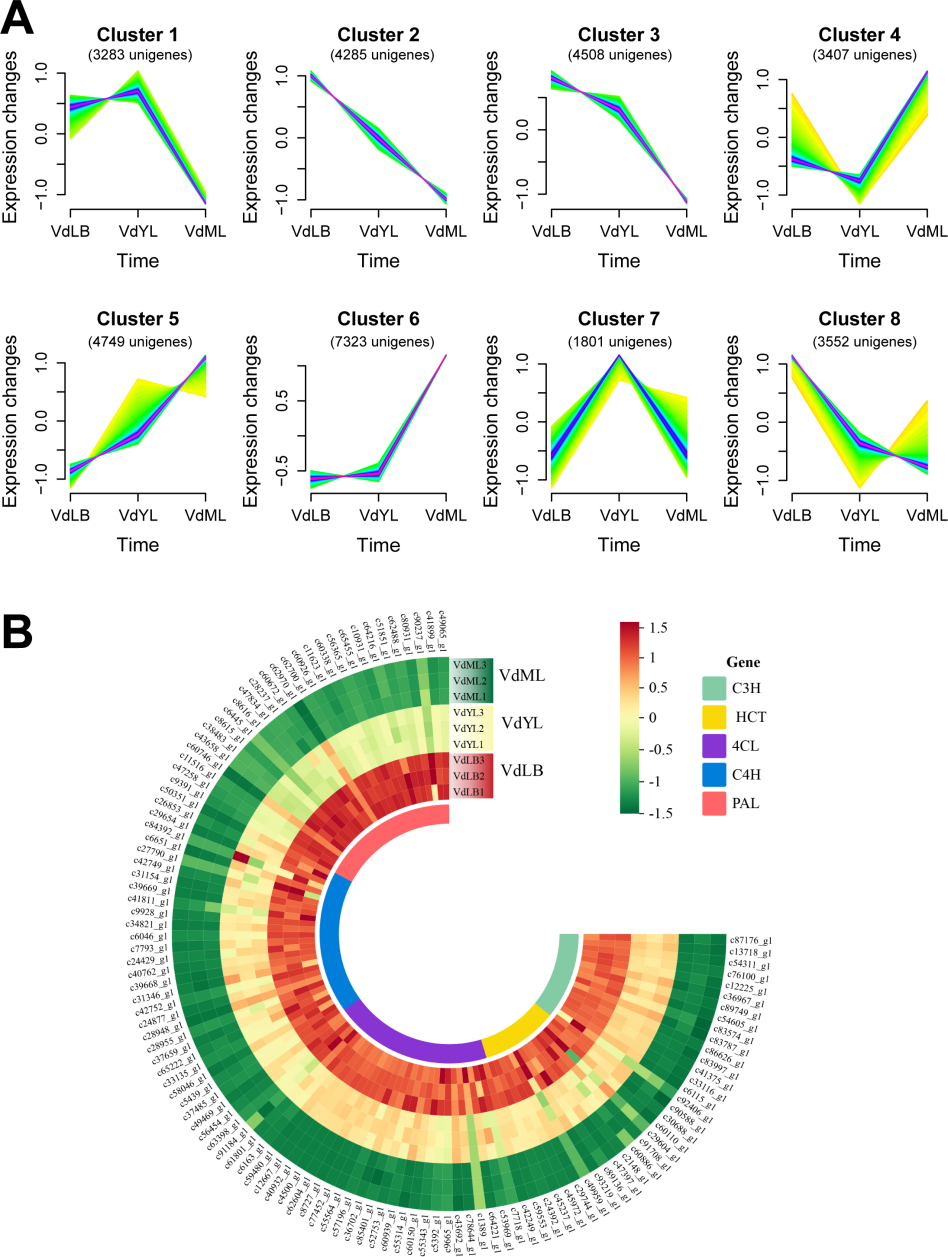
Identification of unigenes associated with CGA biosynthesis based on transcriptomics. **(A)** Expression profiles of all DEGs by Mfuzz clustering analysis. **(B)** Unigenes related to CGA biosynthesis in *V. dunalianum*.

To unveil the unigenes responsible for CGA biosynthesis in *V. dunalianum*, 32,098 DEGs obtained from merging DEGs from the three comparison groups were subjected to gene expression time trend analysis using the Mfuzz package with the Fuzzy C-Means Clustering (FCM) algorithm. The results indicated that 8 gene clusters (**Figure 4A**) were obtained, in which the genes in Cluster2, Cluster3, and Cluster8 exhibited a declining trend in expression abundance as leaf growth and development progressed, aligning with the observed variations in CGA content during leaf tissue development. Hence, a detailed exploration of gene information in the three clusters led to the preliminary identification of 118 potential genes associated with CGA biosynthesis, including 27 *PALs*, 28 *C4Hs*, 31 *4CLs*, 15 *HCTs*, and 17 *C3Hs* (**Figure 4B**).

### 3.5 Characterization of key unigenes for CGA biosynthesis in *V. dunalianum* based on the joint analysis of the transcriptome and proteome

A total of 6,876 (**Supplementary Table S2**) proteins were quantified through quantitative proteomic analysis of VdLB, VdYL, and VdML samples based on the 4D label-free technique. A comparison analysis was carried out on the proteomes of leaves at three typical developmental stages, with |log_2_FoldChange|>1 and p-value < 0.05 used as the criteria for identifying DEPs between each pair of comparison groups. It was observed that 142 same DEPs were found to be present in all three comparison groups (**Supplementary Figure S3**).

**Figure 5:**
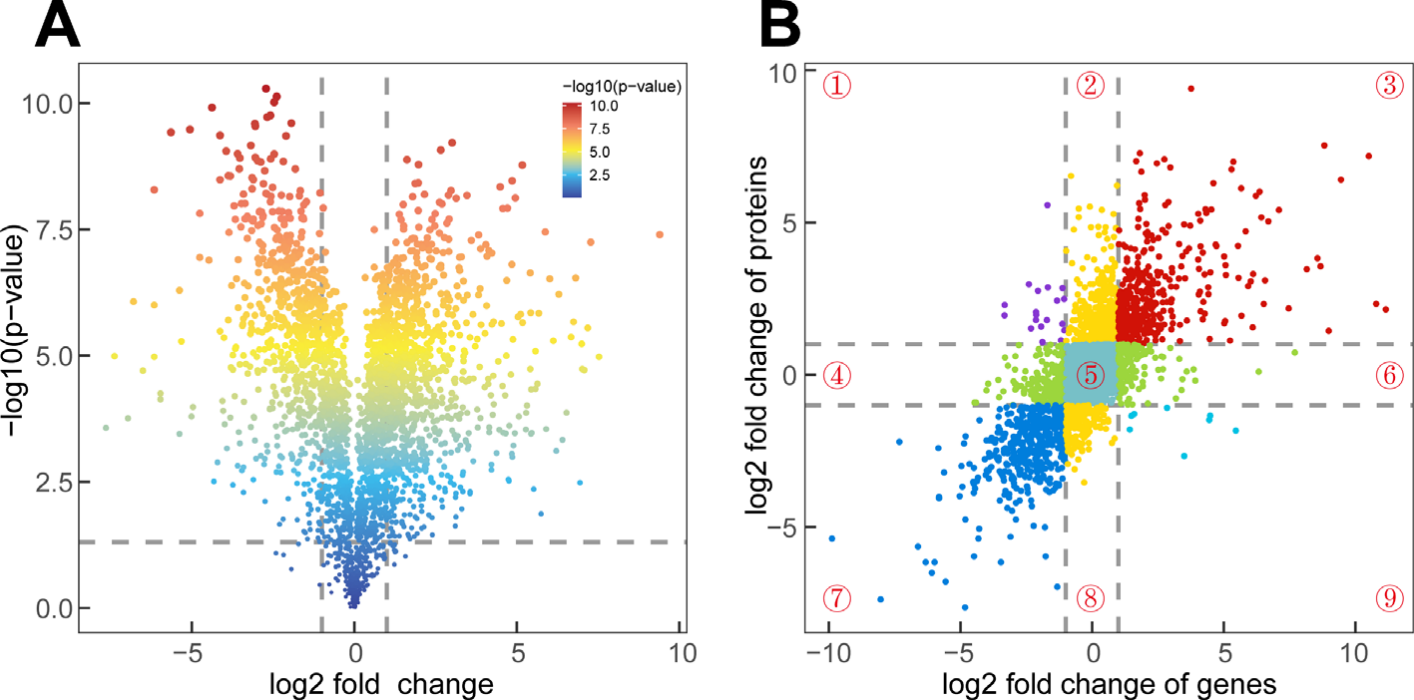
Joint analysis of the transcriptome and proteome. **(A)** Volcano plots of the DEPs in VdML vs VdLB. Each dot represents a protein. **(B)** Scatter plot of 9-quadrant association analyses of mRNA and protein levels from log_2_FoldChange in VdML vs VdLB. Each dot represents a gene or protein. Number ①–⑨: quadrant number.

In the “VdYL vs VdLB” group, 622 DEPs were identified, and in the “VdML vs VdYL” group, 1,457 DEPs were detected. Similar to the results of the transcriptomic comparisons, the “VdML vs VdLB” group demonstrated the highest count of DEPs, amounting to 1,481 DEPs. When VdML was employed as the reference, 871 proteins exhibited an upregulation in their expression levels, while 610 proteins displayed a downregulation (**Figure 5A**). Consequently, a joint analysis of DEGs and DEPs in the “VdML vs VdLB” group can be conducted to identify key genes involved in CGA biosynthesis in *V. dunalianum*.

A nine-quadrant analysis of the VdLB and VdML groups was conducted in R to show the relationship between RNAs and proteins (**Figure 5B**). The log_2_FoldChange values from RNA-Seq were set on the X-axis, while the proteomic log_2_FoldChange values were placed on the Y-axis, with the expression changes of genes and proteins detailed into nine distinct patterns, each quadrant representing a specific combination of expression changes. In this study, a total of 2,748 quantified proteins/genes were categorized into nine quadrants. The results indicate that the significant enrichment observed in quadrant 5 implies a common expression of these 934 genes and proteins. It is worth noting that, the 404 genes and proteins in quadrant three and the 444 genes and proteins in quadrant seven exhibited the same expression patterns, and subsequent KEGG enrichment analysis on the genes and proteins in quadrant three revealed significant enrichment across “Ribosome”, “Flavonoid biosynthesis”, and “Phenylpropanoid biosynthesis” pathways. The CGA is a collaborative product of both the “Phenylpropanoid biosynthesis” pathway and the “Flavonoid biosynthesis”. The high levels of CGA in VdLB indicate active secondary metabolism in the early development of *V. dunalianum* leaves, with a relatively higher abundance of genes and proteins involved in both pathways, further confirmed by the KEGG enrichment result. Additionally, genes and proteins from quadrant seven displayed significant enrichment in seven pathways, including “Photosynthesis”, “Nitrogen metabolism”, and “Tryptophan metabolism”. Given the higher CGA content in VdLB compared to VdML, a similar trend in abundance is noted in the 404 genes and proteins of the third quadrant. Consequently, it is essential to search for key genes involved in CGA biosynthesis in these 404 genes and proteins.

**Figure 6:**
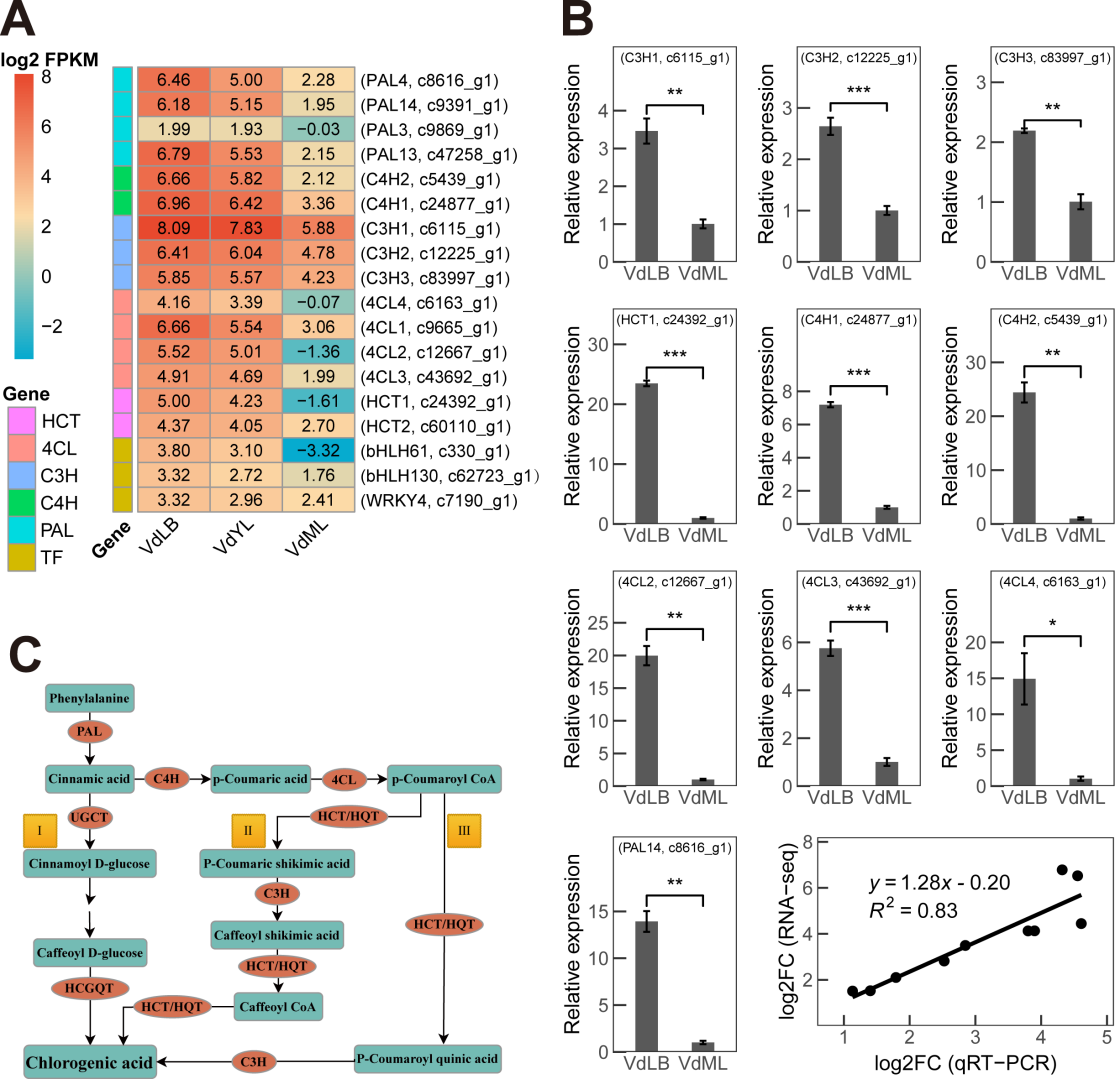
CGA biosynthesis pathway and synthesis genes in *V. dunalianum*. **(A)** The heatmap displays fifteen key unigenes involved in CGA biosynthesis in *V. dunalianum*, along with three potential transcription factors (TF). The numbers represent the log_2_ FPKM values corresponding to the unigenes. **(B)** qRT-PCR validation of the ten key unigenes associated with CGA biosynthesis in *V. dunalianum* under VdLB and VdML. The data represent the mean values ± standard deviation of three biological replicates. Asterisks denote statistical significance based on Student’s t-test (*: *P* < 0.05; **: *P* < 0.01; ***: *P* < 0.001). The final graph illustrates the Pearson correlation between RNA-seq and RT-qPCR data. **(C)** The three CGA biosynthesis pathways in plants. The genes involved in CGA biosynthesis in plants including phenylalanine ammonia lyase (PAL), cinnamate 4-hydroxylase (C4H), 4-coumarate: CoA ligase (4CL), P-coumarate 3-hydroxylase (C3H), Shikimate O-hydroxycinnamoyltransferase (HCT/HQT), UDP-glucose: cinnamate glucosyltransferase (UGCT) and hydroxyl cinnamoyl D-glucose: quinate hydroxycinnamoyl transferase (HCGQT).

Ultimately, through a nine-quadrant analysis and in-depth exploration of genes and proteins annotated as relevant to CGA biosynthesis, our study identified that 5 enzymes responsible for CGA biosynthesis in *V. dunalianum* are encoded by 15 key structural genes, consisting of 4 *PALs*, 2 *4CHs*, 4 *4CLs*, 2 *HCTs*, and 3 *C3Hs*. The expression levels of these key genes at the transcriptional level were observed to be higher in VdLB than in VdYL and VdML (**Figure 6A**). Subsequently, 10 key genes responsible for chlorogenic acid biosynthesis were selected to be subjected to RT-qPCR experiments in VdLB and VdML (**Figure 6B**). The experimental results were consistent with the conclusions drawn from transcriptomic and proteomic analyses, showing higher expression levels in VdLB compared to VdML. The correlation between the results of RT-qPCR and transcriptomic analysis was determined to be 0.83, further validating the reliability of the transcriptomic data.

As reported, the biosynthesis of CGA in plants involves three pathways with the participation of multiple genes (**Figure 6C**) (Kundu and Vadassery, 2019; Wen et al., 2022). Among the biosynthetic pathways of CGA, phenylalanine ammonia lyase (PAL), cinnamate 4-hydroxylase (C4H), 4-coumarate: CoA ligase (4CL), P-coumarate 3-hydroxylase (C3H), shikimic acid/quinic acid hydroxycinnamoyl transferase (HCT), and hydroxycinnamoyl-CoA quinic hydroxycinnamoyl transferase (HQT) are key enzymes in the biosynthesis of CGA. Inducing the expression of these enzymes helps to increase the content of CGA in plants (Chen et al., 2024).

PAL is a key bridge connecting primary metabolism and secondary metabolism. It catalyzes the conversion of phenylalanine to cinnamic acid, acting as a metabolic flux that controls the entry of precursors into the phenolic pathway. It plays an important role in regulating the overall accumulation of phenolic compounds (Clé et al., 2008). C4H is the second key rate-limiting enzyme in the phenylpropanoid pathway. In the presence of oxygen and reduced nicotinamide adenine dinucleotide phosphate (NADPH), the enzyme uses NADPH as an electron donor to catalyze the conversion of trans-cinnamic acid to p-coumaric acid (Cheng et al., 2018; Zhang et al., 2020). 4CL is the main branching point in the cinnamic acid pathway, where it converts cinnamic acid derivatives, including p-coumaric acid, caffeic acid, ferulic acid, and cinnamic acid, into different types of coenzyme A derivatives. These intermediates serve as the basis for the synthesis of phenylpropanoid compounds, such as lignin, CGA, and flavonoids (Chen et al., 2024). C3H is another key enzyme in the chlorogenic acid synthesis pathway. The expression of C3H can also regulate the synthesis of secondary metabolites such as flavonoids and CGA (Li et al., 2016). HCT is a key enzyme in the chlorogenic acid synthesis pathway and can positively regulate the synthesis of chlorogenic acid (Li et al., 2015). HQT is closely linked to HCT. Unlike the substrate versatility of HCT, HQT has substrate specificity and is the final key rate-limiting enzyme involved in the biosynthesis of CGA. It catalyzes the transesterification of caffeoyl-CoA and quinic acid to produce CGA with different structures (Li et al., 2019).

Notably, in the absence of detected HCGQT homologs, which indicate the lack of Pathway I for CGA biosynthesis in *V. dunalianum*, it is inferred that CGA in *V. dunalianum* is likely synthesized via Pathways II and III. As widely recognized, transcription factors play a crucial role in modulating gene expression by regulating transcription regulation domains, leading to the activation or inhibition of target gene transcription. This regulatory function extends to controlling the flux of metabolic pathways, thereby influencing the accumulation of metabolites. Prior research has demonstrated that TabHLH1 directly associates with the promoters of *TaHQT2* and *Ta4CL* via the E-box motif (CATGTG), leading to a direct upregulation of *TaHQT2* and *Ta4CL* gene expression and subsequently influencing the accumulation of CGA (Liu et al., 2021). Moreover, Wang et al. identified the *NtWRKY33a* gene in the *Nicotiana tabacum* genome, suggesting its potential involvement in regulating metabolic flux distribution in the phenylpropanoid pathway, and demonstrated its capability to enhance CGA biosynthesis through overexpression and knockout experiments (Wang et al., 2023). Similarly, the *NtWRKY41A* gene was suggested to promote the biosynthesis of CGA in tobacco (Wang et al., 2022). However, whether other members of the WRKY and bHLH transcription factor families can regulate key enzymes in the CGA biosynthesis pathway remains unknown, and so far, no transcription factor regulating the biosynthesis of CGA has been identified in *V. dunalianum*. Two members of the bHLH transcription factor family, namely bHLH61 encoded by unigene c330_g1 and bHLH130 encoded by unigene c62723_g1, as well as one member (WRKY4, c7190_g1) of the WRKY transcription factor family (**Figure 6A**), were identified through a combined transcriptomic and proteomic analysis in this study. The changing trends in expression levels of these three transcription factors across three developmental stages of *V. dunalianum* leaves and the expression levels of key enzyme genes in the CGA biosynthesis pathway are similar, all gradually decreasing as leaves progress in their growth and development. Further research is required to investigate whether these three transcription factors can be involved in regulating CGA biosynthesis in *V. dunalianum*.

### 3.6 Non-targeted metabolomics analysis of *V. dunalianum* leaves

#### 3.6.1 Metabolic profiling of the three developmental stages of *V. dunalianum* leaves

To explore the dynamic changes in metabolites during *V. dunalianum* leaf development, non-targeted metabolomics analysis was performed using a UHPLC-Q-TOF MS system. A total of 1,457 metabolites (**Supplementary Table S3**) were detected, with 936 metabolites identified under positive ion (POS) mode and 521 metabolites under negative ion (NEG) mode. These metabolites were classified into 11 categories at the superclass level, including “Lipids and lipid-like molecules” (413), “Phenylpropanoids and polyketides” (287), “Organic oxygen compounds” (135), “Benzenoids” (123), “Organoheterocyclic compounds” (119), “Organic acids and derivatives” (71), “Alkaloids and derivatives” (26), “Lignans, neolignans and related compounds” (21), “Nucleosides, nucleotides, and analogues” (20), “Organic nitrogen compound” (20), “Hydrocarbon derivatives” (1). Furthermore, 221 metabolites lack superclass annotation information.

#### 3.6.2 Change of DAMs in *V. dunalianum* leaves during the developmental stage

**Figure 7:**
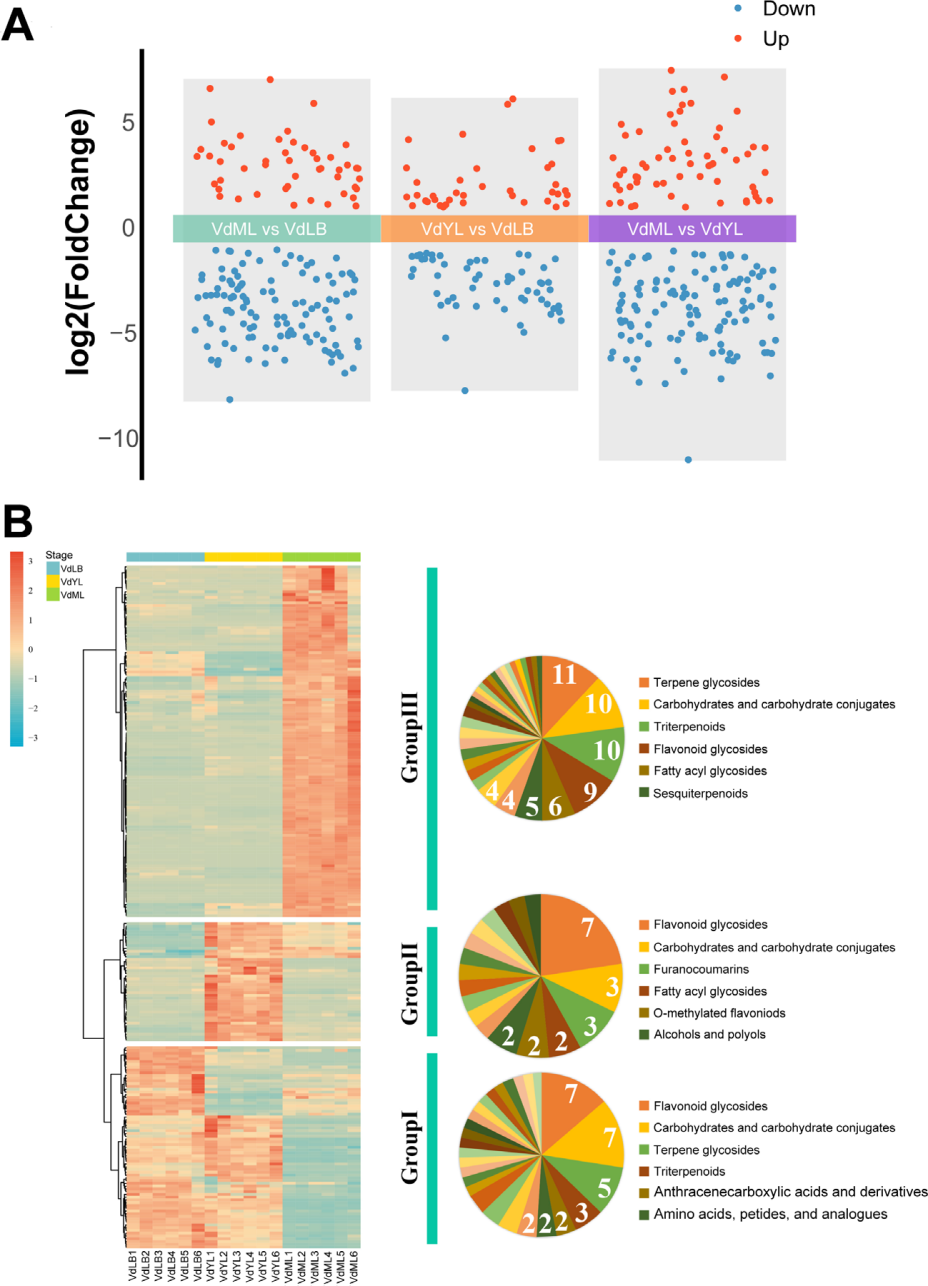
Non-targeted metabolomics analysis of leaf development in *V. dunalianum.* **(A)** Number of DAMs among the different comparison groups. **(B)** Changes in the DAMs during *V. dunalianum* leaves development.

The DAMs between the three different comparison groups (“VdML vs VdLB”, “VdYL vs VdLB” and “VdML vs VdYL”) were then analyzed based on a VIP value of >1, Fold Change of >2 or < 0.5 and p-value of < 0.05. For each comparison group, the upregulated metabolite amounts ranged from 39 to 61, whereas the downregulated metabolite amounts ranged from 56 to 124 (**Figure 7A**). Significantly, in comparison to VdML, the upregulated metabolites exhibit higher contents during both VdLB and VdYL, making a significant contribution to the revelation of Quezui Tea’s medicinal and nutritional value.

To delve deeper into the metabolic composition underlying the nutritional and medicinal value of Quezui Tea, DAMs from three comparison groups were merged, resulting in a total of 243 DAMs. Among these, 174 DAMs were categorized into 58 subclasses, while 69 DAMs lacked subclass annotation information. Subsequently, a heatmap of all the DAMs was generated using R, and the trends in content variation were mainly clustered into three groups (**Figure 7B**; **Supplementary Table S4**). The majority of DAMs in Group I exhibit higher content during the VdLB and VdYL, with a subsequent decrease in content during the VdML. Among DAMs with subclass classification information, Group I is principally composed of “Flavonoid glycosides” and “Carbohydrates and carbohydrate conjugates”, followed by “Terpene glycosides”. In Group II, DAMs content displays a pattern of initially increasing and then decreasing, with the highest levels observed during the VdYL. Among DAMs with subclass classification information, “Flavonoid glycosides” are the most prevalent. In Group III, DAMs levels show minimal changes during the VdLB and VdYL and are comparatively lower than in VdML. However, the DAMs levels rapidly reach their peak in VdML. Among DAMs with subclass classification information, this group chiefly comprised “Terpene glycosides”, “Carbohydrates and carbohydrate conjugates”, “Triterpenoids”, and “Flavonoid glycosides”.

It can be concluded that during the growth and development of *V. dunalianum* leaves, especially during the VdLB and VdYL, the accumulation of “Flavonoid glycosides” in the category of flavonoid compounds is of particular importance. As a co-product of the phenylpropanoid biosynthesis pathway and the flavonoid biosynthesis pathway, the high content characteristic of CGA in VdLB and VdYL also implies the presence of abundant flavonoid compounds in these stages. Flavonoid compounds consistently occupy a crucial position as essential metabolites and extracts in plants, serving not only to respond to both biotic and abiotic stress factors during plant growth and development, including antioxidant, UV-protection, pathogen-resistance, and cold-tolerance properties but also to offer various nutritional and health benefits for human because of their broad medicinal value. In addition, the types and content of flavonoid compounds are important criteria for assessing the quality of tea. Hence, the exploration of flavonoid compounds in Group I and Group II is extremely important. We found that Group I contains Procyanidin C1, Baicalein, Isoliquiritin, Eriocitrin, Tiliroside, and Luteolin 7-*O*-rutinoside, while Group II contains Guaijaverin, Kaempferol 3-*O*-arabinoside, Nevadensin, Eupatilin, Delphinidin 3-glucoside, Naringenin, Cyanidin 3-*O*-glucoside, Quercitrin, Isoquercitrin, and Tulipanin. Previous studies have shown that these flavonoid compounds possess miscellaneous medicinal activities, including anticancer (Wang et al., 2016; Koteswari et al., 2020), antidiabetic (Goto et al., 2012), antioxidant and anti-inflammatory (Jung et al., 2018), antidepressant (Wang et al., 2008), neuroprotective (Ma et al., 2016), and more. Notably, Zandi et al. have shown that baicalein, as an effective component of traditional Chinese medicine, has significant inhibitory activity against COVID-19 (Zandi et al., 2021).

Historically, research on *V. dunalianum* has been limited to the application of traditional plant chemical analysis methods to study certain specific natural products. With no reported research on the composition of metabolic profiles in *V. dunalianum* leaves and the dynamic changes in these profiles during growth and development, the utilization of additional value from *V. dunalianum* has been hindered. Therefore, an in-depth understanding of the composition and changes in metabolites during the development of *V. dunalianum* is of great significance for the functional enhancement and quality evaluation of Quezui tea. Through non-targeted metabolomics analysis, our study achieved the most comprehensive and extensive detection of metabolites in *V. dunalianum* leaves, thereby addressing the limitations of previous research and providing rich metabolic profile information. Additionally, our study marks the first to report the accumulation and variations of DAMs during the *V. dunalianum* leaf development. We found that the predominant accumulation of DAMs during the early development of *V. dunalianum* leaves was associated with flavonoid compounds, with a particular focus on flavonoid glycosides, and provided a detailed account of flavonoid compounds in VdLB and VdYL. These findings offer scientific insights into the nutritional, medicinal, and industrial potential of *V. dunalianum* and provide a robust data foundation for establishing a quality evaluation system for Quezui Tea. Additionally, these results highlight the potential of *V. dunalianum* as a daily dietary supplement rich in phenolic compounds.

In conclusion, our multi-omics analysis provides a comprehensive dataset with significant implications for network pharmacology. This holistic approach not only deepens our understanding of the metabolic profile of *V. dunalianum* but also offers a valuable resource for exploring its nutritional and medicinal values. The insights gained from this analysis could potentially inform the development of a quality evaluation system for Quezui Tea, thereby enhancing our ability to harness the plant’s therapeutic potential.

## 4 Conclusion

In summary, our study identified 15 key structural genes influencing CGA biosynthesis in *V. dunalianum*, along with three potential transcription factors that may regulate its biosynthesis. This has clarified the biosynthetic pathway of CGA in *V. dunalianum* and provided evidence for unraveling the regulatory network of CGA biosynthesis. These findings provide abundant data for the industrialization of *V. dunalianum* production and the utilization of *V. dunalianum* as a natural botanical resource for extracting CGA. Furthermore, our untargeted metabolomics analysis provides the first extensive report on the metabolic profile of *V. dunalianum*. It reveals that the VdLB and VdYL of *V. dunalianum* are used in the production of Quezui Tea due to their higher flavonoid content, which provides a valuable dataset for deeper exploration of its nutritional and medicinal value, and the development of a quality evaluation system. Our study has provided comprehensive and in-depth molecular information for the advancement of molecular breeding in *V. dunalianum*. We believe that this information will play a pivotal role in *V. dunalianum* breeding, offering strong support for the development of high-yielding, stress-resistant, and high-quality new *V. dunalianum*.

## Data Availability

The raw data of RNA-Seq underlying this study are openly available in the SRA database under project accession PRJNA1037676 (https://www.ncbi.nlm.nih.gov/sra/PRJNA1037676). Proteome and metabolome data has been uploaded as **Supplementary Tables S2**, **S3**. Any supporting data to this article can be reasonably requested from corresponding authors.

## Supplementary material

Supplementary Figure 1 Total ion chromatograms of quality control samples. **(A)** Positive ion mode. **(B)** Negative ion mode.

Supplementary Figure 2 Venn diagram of DEGs among VdML vs VdYL, VdYL vs VdLB, and VdML vs VdLB.

Supplementary Figure 3 Venn diagram of DEPs among VdML vs VdYL, VdYL vs VdLB, and VdML vs VdLB.
