# Supplementary figures and images for: Revealing the dynamic changes of metabolites and molecular mechanisms of chlorogenic acid accumulation during the leaf development of Vaccinium dunalianum based on multi-omic analyses
Source: Front Plant Sci. 2024 Oct 31;15:1440589. doi: 10.3389/fpls.2024.1440589 (PMC11560443; doi:10.3389/fpls.2024.1440589)

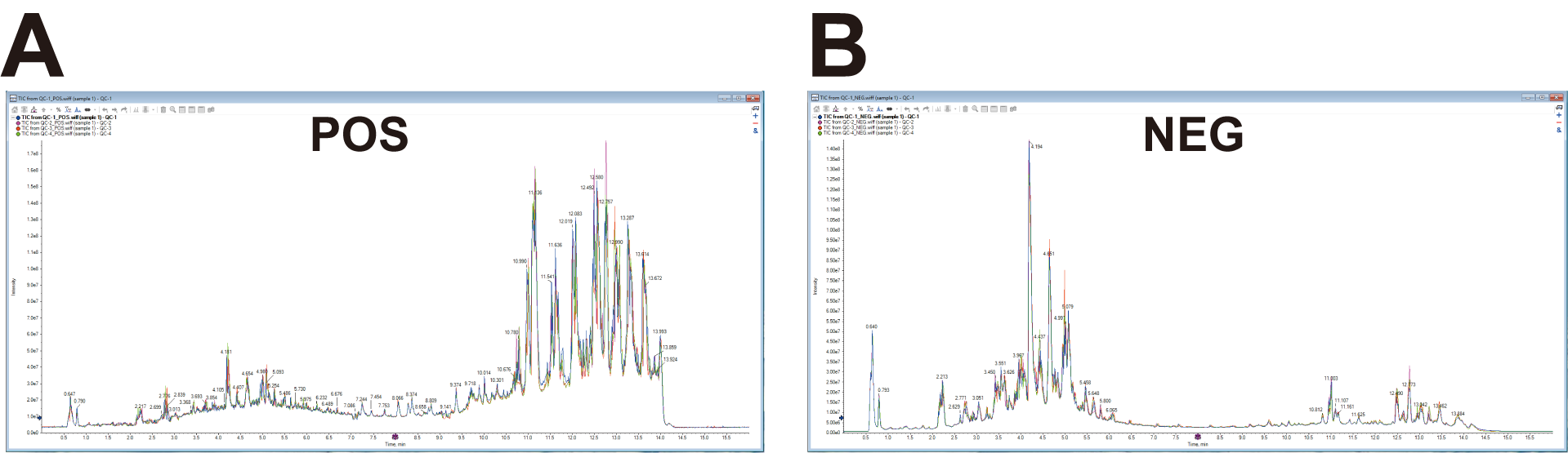

Supplement: Supplementary Figure 1 — Total ion chromatograms of quality control samples. (A) Positive ion mode. (B) Negative ion mode. [file Image1.tif]

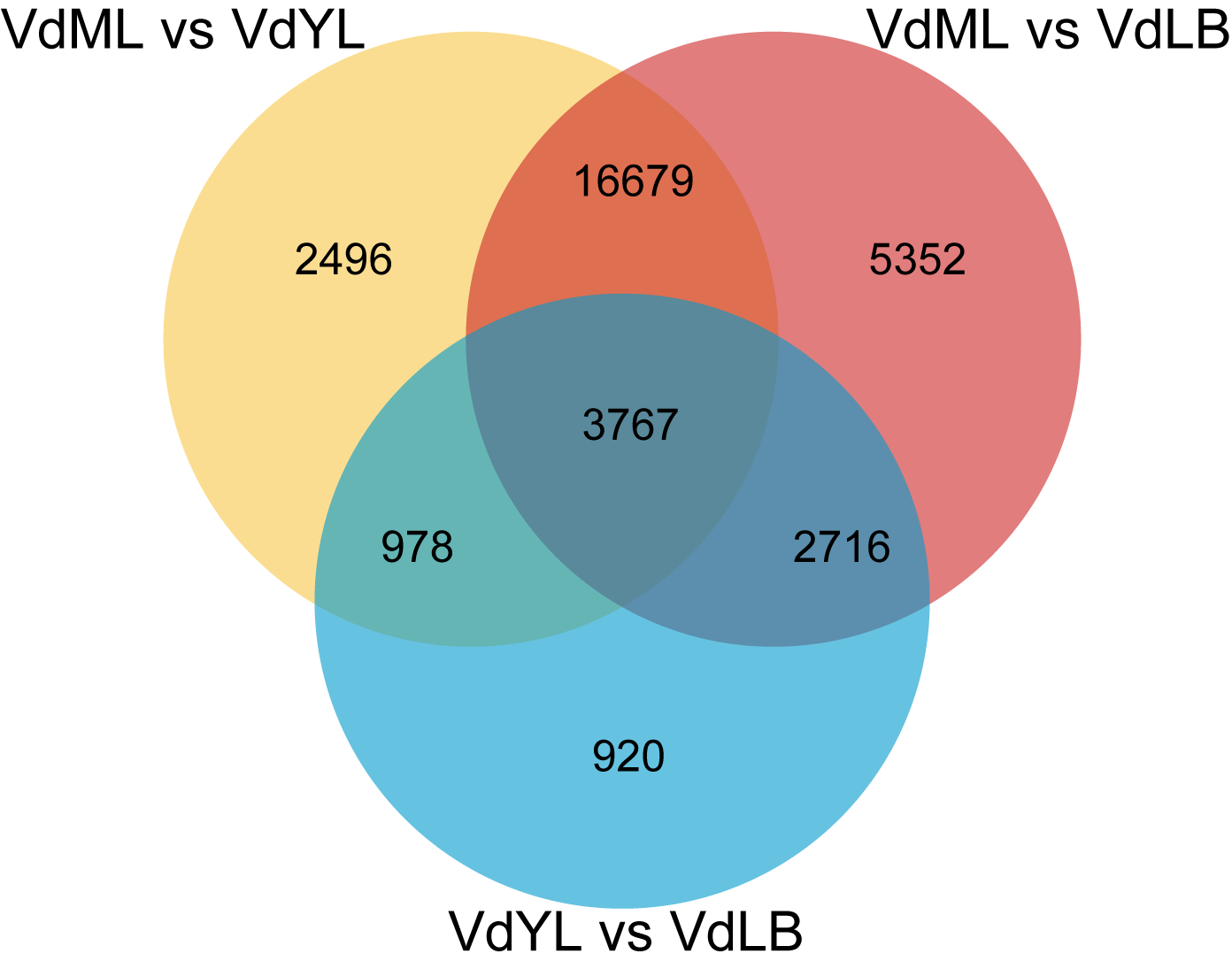

Supplement: Supplementary Figure 2 — Venn diagram of DEGs among VdML vs VdYL, VdYL vs VdLB, and VdML vs VdLB. [file Image2.tif]

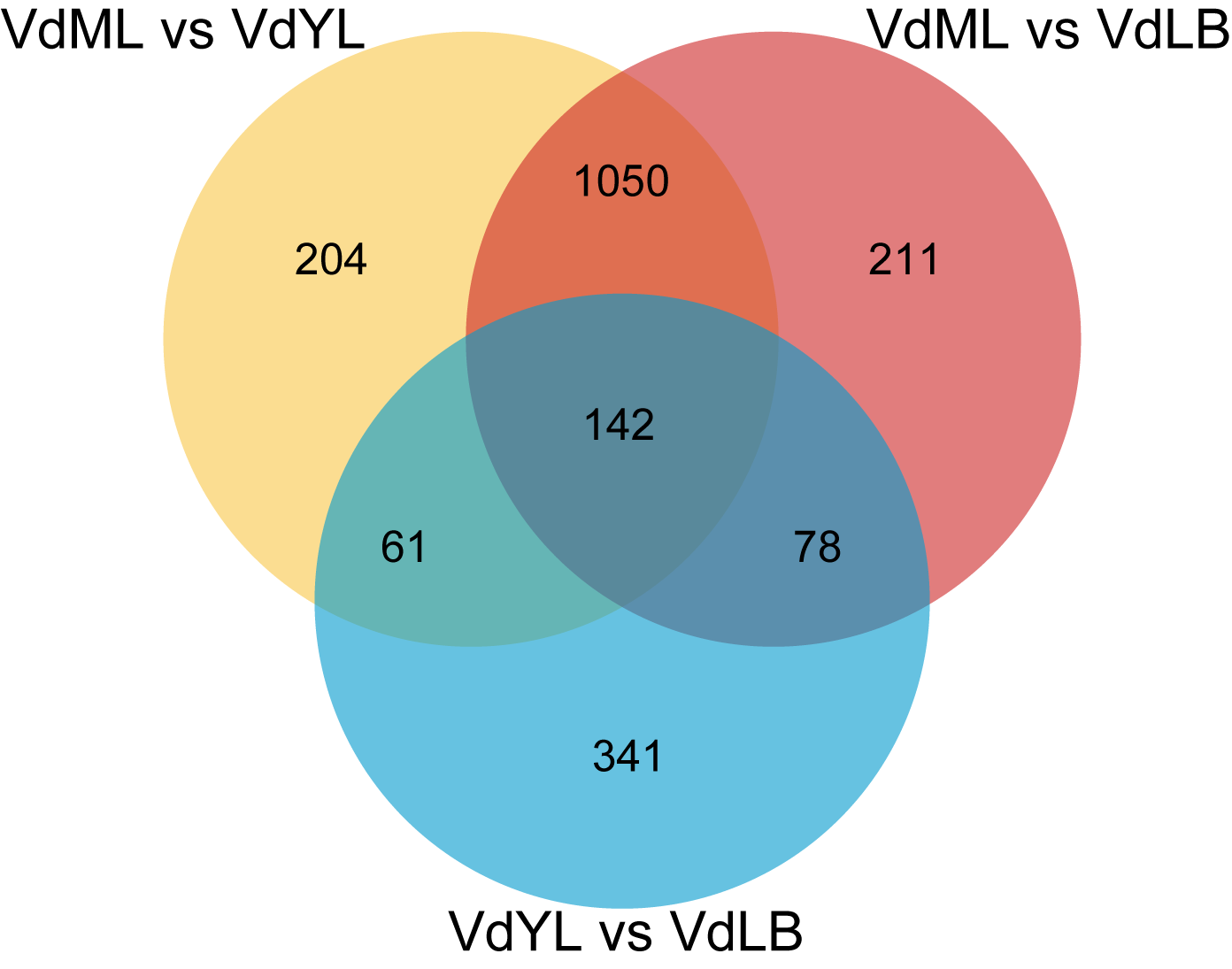

Supplement: Supplementary Figure 3 — Venn diagram of DEPs among VdML vs VdYL, VdYL vs VdLB, and VdML vs VdLB. [file Image3.tif]
